# Effects of sex differences on scapular motion during arm elevation

**DOI:** 10.1051/sicotj/2015004

**Published:** 2015-06-08

**Authors:** Takashi Nagamatsu, Yoshihiro Kai, Masafumi Gotoh, Kazuya Madokoro, Naoto Shiba

**Affiliations:** 1 Department of Physical Therapy, Fukuoka Rehabilitation College 3-29-17 Hakataekimae Hakata-ku, Fukuoka-si, Fukuoka Japan; 2 Faculty of Health Science, Kyoto Tachibana University 34 Yamada-cho Oyake Yamashina-ku Kyoto-si, Kyoto Japan; 3 Department of Orthopedic Surgery, Kurume University Medical Center 155-1 Kokubu-machi Kurume-si, Fukuoka Japan; 4 Kurume University School of Medicine Graduate School 67 Asahi-machi Kurume-si, Fukuoka Japan; 5 Department of Orthopedic Surgery, Kurume University 67 Asahi-machi Kurume-si, Fukuoka Japan

**Keywords:** Scapula, Kinematics, Sex differences, Electromagnetic tracking device, Dynamic analysis

## Abstract

*Introduction*: Scapular motion during arm elevation is frequently evaluated in patients with shoulder disorders because it provides clinically useful information. With the development of measurement devices and improvement in accuracy, comparisons under various conditions have recently been reported. However, in most of these reports, the subjects examined were limited to males, or a mixed population of males and females. Only a few reports have described sex differences. In the current study, we performed three-dimensional dynamic analysis of arm elevation and investigated whether there is a sex difference in scapular motion.

*Methods*: Subjects included 18 healthy adult males (18 shoulders) and 19 healthy adult females (19 shoulders). Thirty-seven shoulders were on the dominant side. The age range was 20.5 ± 0.03 years. Subjects performed scapular plane arm elevation, and kinematic data were recorded using an electromagnetic tracking device. Scapular upward rotation and internal rotation angles and the posterior tilt angle accompanying arm elevation were calculated from recorded data. Changes in each angle during scapular motion were recorded according to sex.

*Results*: There were sex differences in scapular upward rotation and internal rotation angles. The upward rotation angle was significantly greater in males, whereas the internal rotation angle was significantly greater in females. No sex differences were noted in the scapular posterior tilt angle.

*Discussion*: Findings of this study may serve as basic data for scapular motion during scapular plane elevation in healthy males and females. In addition, it is necessary to evaluate and treat the shoulder while taking sex differences in scapular movement into consideration.

## Introduction

Dynamic analysis of the shoulder joint during arm elevation was introduced as the scapulohumeral rhythm (SHR) by Codman [2], and later, Inman et al. [9] reported that the ratio of movement of humeral elevation and scapular upward rotation in forward and lateral elevations was 2:1 in healthy subjects. Dynamic analyses of the shoulder joint using radiography and goniometer measurements and three-dimensional analysis using a three-dimensional electromagnetic position measurement device have been performed, and SHR during scapular plane arm elevation has been reported to be 1.9–2.4:1, similar to that reported by Inman et al. [4, 12, 13].

Three-dimensional dynamic analyses of the scapula have recently been performed under various conditions, in addition to SHR analysis in healthy subjects, such as comparisons between dominant and nondominant sides [20], between voluntary and passive movements [6, 16], and between uni- and bilateral elevations [3]. However, subjects were limited to males, or the sex difference was limited in most of these studies, and no report described differences between the sexes. Therefore, the objective of this study was to investigate sex differences in scapular motion during arm elevation.

## Materials and methods

### Subjects and outcome measures

The study was approved by the Ethics Committee of Kurume University (#09078), and written consent for participation was obtained from all subjects.

Subjects included 18 healthy males and 19 healthy females (18 and 19 shoulders on the dominant side, respectively). The mean height, body weight, and age were 172 ± 5.5 cm, 64.6 ± 4.5 kg, and 20.9 ± 1.4 years in the male group, respectively, and 157.9 ± 5.2 cm, 52.5 ± 8.3 kg, and 20.5 ± 0.9 years in the female group, respectively. The absence of complaints, trauma, or history of shoulder disorders was confirmed before inclusion in the study. Subjects with hyperlaxity were excluded from this study. Detailed data are presented in Table 1.

**Table 1.** BMI = body mass index; *SD* = standard deviation.

	Male (*n* = 18)		Female (*n* = 19)		*p* value
	Mean	*SD*	Mean	*SD*	
Age (y)	20.9	1.4	20.5	0.9	0.34
Height (cm)	172.0	5.5	157.9	5.2	<0.01
Weight (kg)	64.6	4.5	52.5	8.7	<0.01
BMI (kg/m^2^)	21.9	1.7	21.0	2.7	0.26

During the movement task, subjects were instructed to elevate their arm to the maximum level in the scapular plane (30° anterior to the frontal plane) from a dropped position, which took 3 seconds in a sitting position. Subjects sufficiently practiced the movement task to prevent variation in the elevation speed before measurement; thereafter, two measurements were performed. Kinematic data were collected during the task using an electromagnetic tracking device, LIBERTY (Polhemus, Vermont, USA), and Motion Monitor software^®^ version 8.43 (Innovative Sports Training., Inc., Chicago, IL). The system comprised a transmitter, seven sensors (receivers), a stylus (digitizer), and a system unit, and the accuracy of angle direction has been reported to be 1.3° [14]. The root mean square error generated due to skin motion artifacts is less than 9.4° when the humerus elevation is 120° or smaller [10]. The transmitter generates a low-frequency electromagnetic field detected by each sensor. Data were collected at a 120-Hz sampling rate. The global coordinate system was established by a transmitter mounted on a rigid wooden base frame and aligning it with the cardinal planes of the body. Electromagnetic sensors were attached to the sternum, acromion, and humerus on the dominant side. The bony landmarks were palpated and digitized while the subject sat on a plastic chair and relaxed the arm to the side of the body. The landmarks were chosen in accordance with the International Society of Biomechanics (ISB) [17]: the spinous processes of the 7th cervical and 8th thoracic vertebrae, suprasternal notch, and xiphoid process were the thoracic landmarks, the glenohumeral joint rotation center (estimated by the rotation method) and medial and lateral epicondyles were the humeral landmarks, and the acromial angle, root of the spine of the scapula, and the inferior angle were the scapular landmarks.

Using the recorded three-dimensional data of each segment, the arm elevation, scapular upward rotation, internal rotation, and posterior tilt angles were calculated using the Euler angle rotation sequence recommended by ISB.

### Statistical analysis

Statistical analysis was performed using PASW Statistics for Windows version 17.0 (SPSS Japan, Tokyo, Japan). Concerning calculated angle data, 20–120° arm elevation was regarded as the analytical range, and scapular movement was analyzed at every 10° elevation. Regarding the inter-rater reproducibility of the three scapular movement angle measurements, the intraclass correlation coefficient (type 1, 1) was calculated. For inter-sex comparison of scapular upward rotation and internal rotation angles and the posterior tilt angle at each arm elevation angle, two-way analysis of variance (sex × arm elevation angle) was used. When a significant interaction was detected between the sexes and arm elevation angle, the influence of the sex was further evaluated at each arm elevation angle using the Bonferroni post hoc test. The significance level was set at less than 5% in all analyses.

## Results

ICC (1, 1) of scapular upward rotation was 0.953 (95% confidence interval: 0.939–0.964), internal rotation was 0.667 (95% confidence interval: 0.585–0.736), and posterior tilt was 0.928 (95% confidence interval: 0.906–0.945).

### Scapular upward rotation

**Figure 1.**
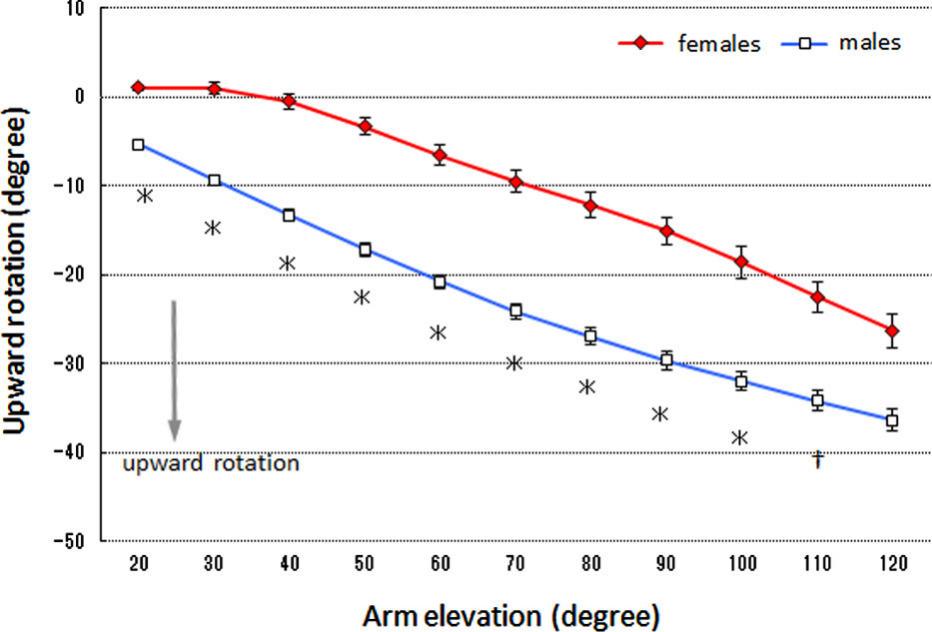
Mean scapular upward rotation. The error bars represent the standard error of the mean. ^*^Significant difference (*p* < 0.01) between groups. ^†^Significant difference (*p* < 0.05) between groups.

A significant interaction was detected between the sexes and arm elevation angle (*df* = 1.56, *F* = 10.48, *p* < 0.001). In addition, a main effect of the sexes was noted (*df* = 1, *F* = 36.73, *p* < 0.001). Regarding multiple comparison, the scapular upward rotation angle was significantly greater in the male group than in the female group within the arm elevation range of 20–100° (*p* < 0.01 for 20–100° elevation, *p* < 0.05 for 110° elevation) (Figure 1).

### Scapular internal rotation

**Figure 2.**
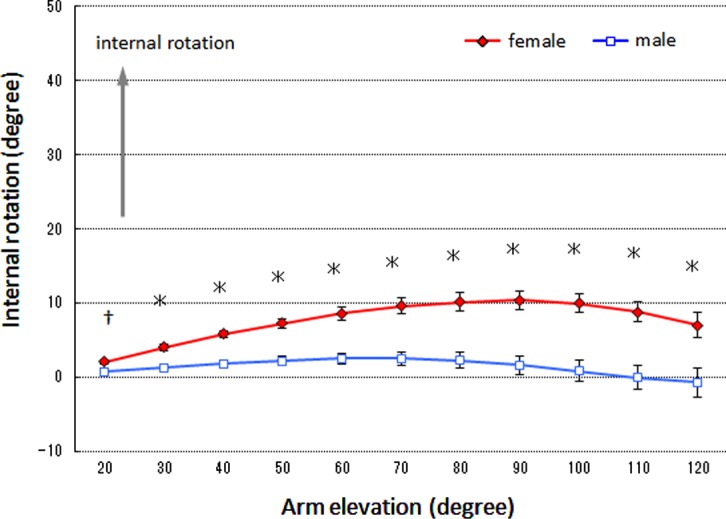
Mean scapular internal rotation. The error bars represent the standard error of the mean. ^*^Significant difference (*p* < 0.01) between groups. ^†^Significant difference (*p* < 0.05) between groups.

A significant interaction was noted between the sexes and arm elevation angle (*df* = 1.41, *F* = 8.43, *p* < 0.01). Regarding multiple comparison, the scapular internal rotation angle was significantly greater in the female group than in the male group within the arm elevation range of 20–120° (*p* < 0.01 for 30–120° elevation, *p* < 0.05 for 20° elevation) (Figure 2).

### Scapular posterior tilt

**Figure 3.**
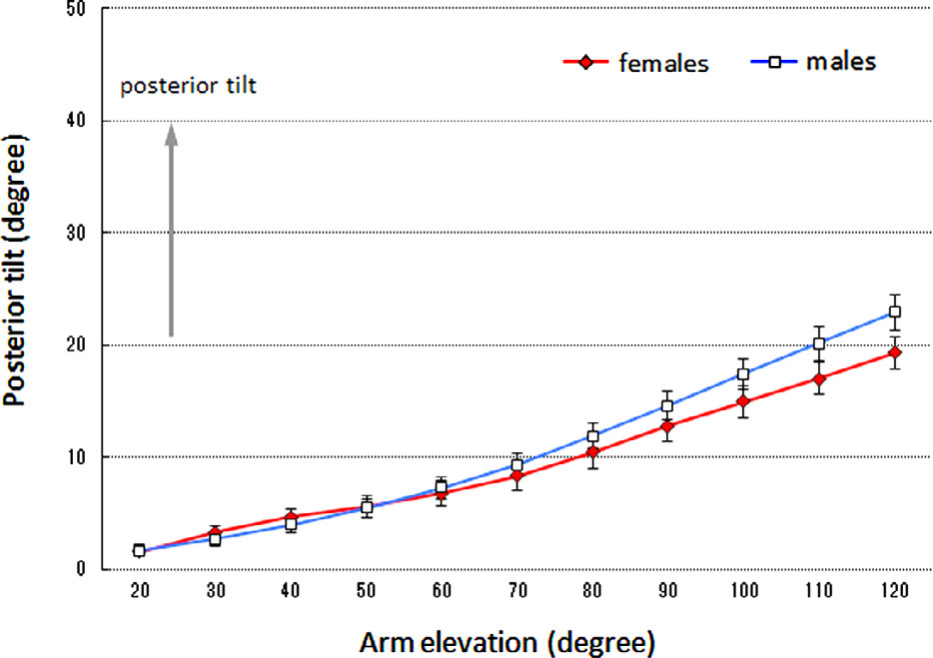
Mean scapular posterior tilt. The error bars represent the standard error of the mean. There was no significant difference between the sexes (*p* = 0.43).

No significant interaction was noted between the sexes and arm elevation angle (*df* = 1.71, *F* = 3.15, *p* = 0.058), and no main effect of the sexes was noted (*df* = 1, *F* = 0.641, *p* = 0.429) (Figure 3).

## Discussion

Dynamic analysis of the shoulder joint during arm elevation has been investigated under various conditions and in various types of subjects with the development of analytical devices, but there has been no report describing sex differences. Therefore, the current study was performed and it clarified that scapular upward rotation and internal rotation angles during arm elevation were different for each sex. The upward rotation angle was significantly greater in males, and the internal rotation angle was significantly greater in females.

Yano et al. [18] performed three-dimensional dynamic analysis of scapular motion during arm elevation in healthy subjects and observed that there were two scapular upward rotation patterns: the scapula rotated downward in the early phase of elevation and then rotated upward (glenohumeral [GH] type), whereas upward rotation occurred from the early phase of elevation in the others (scapulothoracic [ST] type). The upward rotation angle was greater in the ST type compared with the GH type. They performed a follow-up study in which electromyography of the muscles around the shoulder was performed in each type, and the trapezius and serratus anterior muscle activity levels were lower in the GH type compared with the ST type [19]. In the current study, the upward rotation was similar to the GH type in the female group and ST type in the male group. The decreased scapular upward rotation angle observed in the female group may have been due to differences in trapezius and serratus anterior muscle activity.

Ogston and Ludewig [15] compared scapular movement during 10–120° scapular plane arm elevation between multidirectional instability (MDI) patients and control subjects, and they observed that the scapular upward rotation angle was significantly smaller, and the scapular internal rotation angle was greater in all MDI patients during the elevation phase. In the current study the upward rotation angle was smaller in females at all elevation angles, excluding 120°, and the internal rotation angle was greater in females at all elevation angles, which is similar to their findings. No person with shoulder instability was included in the present study, but a higher incidence of generalized joint laxity in females was reported [5, 7, 11]. In addition, in a survey of Asians of the same age group as our subjects, the incidence of generalized joint laxity was 25.4% and 38.5% in males and females, respectively, suggesting that the presence or absence of this laxity was involved in the current results [1]. A follow-up study with a generalized joint laxity test and electromyographic evaluation is necessary.

In clinical relevance, we suggest that the sex differences should be taken into consideration when studies on scapular motion are performed; otherwise, the data evaluation may not be properly determined.

There are several limitations of this study. First, the age range of subjects was narrow and did not correspond to adult males and females in the general population. Habechian et al. [8] reported that there were differences in scapular movement and the SHR during arm elevation between an adult group with a mean age of 35 years and a young group with a mean age of 9 years. Age-related changes in posture, muscle strength, and range of motion of joints may lead to changes in scapular movement in adults; therefore, further investigation by generation is needed. Second, only scapular plane elevation was analyzed; therefore, further investigation of sex differences in scapular movement during forward and lateral elevations is necessary.

## Conclusions

We measured scapular movement during scapular plane arm elevation using an electromagnetic tracking device to investigate the presence of differences between males and females. The upward rotation angle was significantly smaller, and the internal rotation angle was significantly greater in females, showing that the scapular upward and internal rotation patterns were different. These findings may serve as basic data and aid in scapular movement analysis of scapular plane arm elevation and may be useful for functional evaluation of the shoulder joint in clinical practice and disease research.

## Conflict of interest

None of the listed authors received any funding for the work.

None of the authors, their immediate families, and any research foundation with which they are affiliated received any financial payments or other benefits from any commercial entity related to the subject of this article.
